# Supplementary material for: A quantitative pipeline for whole-mount deep imaging and analysis of multi-layered organoids across scales
Source: eLife. 2026 May 22;14:RP107154. doi: 10.7554/eLife.107154 (PMC13197165; doi:10.7554/eLife.107154)
Supplement: MDAR checklist [file elife-107154-mdarchecklist1.pdf]

Materials Design Analysis Reporting (MDAR)
Checklist for Authors

The MDAR framework establishes a minimum set of requirements in transparent reporting mainly applicable to studies in the life sciences.

eLife asks authors to provide detailed information within their article to facilitate the interpretation and replication of their work. Authors can also upload supporting materials to comply with relevant reporting guidelines for health-related research (see EQUATOR Network), life science research (see the BioSharing Information Resource), or animal research (see the ARRIVE Guidelines and the STRANGE Framework; for details, see eLife's Journal Policies). Where applicable, authors should refer to any relevant reporting standards materials in this form.

For all that apply, please note where in the article the information is provided. Please note that we also collect information about data availability and ethics in the submission form.

Materials:

**Newly created materials**	**Indicate where provided: section/figure legend**	**N/A**
The manuscript includes a dedicated "Data and Code Availability" providing transparent disclosure about availability of newly created materials including details on how materials can be accessed and describing any restrictions on access.	In this manuscript, "materials" are **software + trained models + datasets** and they're covered by the data/code statement, Materials and Methods + Data/Code availability statements: Tapenade https://github.com/GuignardLab/tapenade. analysis pipeline and associated napari plugins are openly available (GitHub) https://github.com/GuignardLab/napari-manual-registration, and https://github.com/GuignardLab/napari-tapenade-processing and https://github.com/GuignardLab/napari-spatial-correlation-plotter<br>Custom-trained StarDist3D models and the datasets used for training are https://zenodo.org/records/14748083	

**Antibodies**	**Indicate where provided: section/figure legend**	**N/A**
For commercial reagents, provide supplier name, catalogue number and RRID, if available.	Materials and Methods: Immunostaining/Antibodies (table 2) lists supplier, catalog number, host species and dilution.	

**DNA and RNA sequences**	**Indicate where provided: section/figure legend**	**N/A**
Short novel DNA or RNA including primers, probes: Sequences should be included or deposited in a public repository.		N/A

**Cell materials**	**Indicate where provided: section/figure legend**	**N/A**
Cell lines: Provide species information, strain. Provide accession number in repository OR supplier name, catalog number, clone number, OR RRID.	Materials and Methods: Gastruloid generation and cell lines. Mouse embryonic stem cell lines used include T-Bra-GFP/NE-mKate2; E-cad-GFP/Oct4-mCherry; E14Tg2a.4 (ATCC); and H2B-GFP (gift from Kat Hadjantonakis). All cell lines were tested mycoplasma negative by qPCR.	
Primary cultures: Provide species, strain, sex of origin, genetic modification status.		N/A

**Experimental animals**	**Indicate where provided: section/figure legend**	**N/A**
Laboratory animals or Model organisms: Provide species, strain, sex, age, genetic modification status. Provide accession number in repository OR supplier name, catalog number, clone number, OR RRID.	N/A (no *in vivo* vertebrate animal experiments; gastruloids derived from established mouse ESC lines).	N/A
Animal observed in or captured from the field: Provide species, sex, and age where possible.		N/A

**Plants and microbes**	**Indicate where provided: section/figure legend**	**N/A**
Plants: provide species and strain, ecotype and cultivar where relevant, unique accession number if available, and source		N/A

(including location for collected wild specimens).		
Microbes: provide species and strain, unique accession number if available, and source.		N/A

**Human research participants**	**Indicate where provided: section/figure legend) or state if these demographics were not collected**	**N/A**
If collected and within the bounds of privacy constraints report on age, sex, gender and ethnicity for all study participants.		N/A

Design:

**Study protocol**	**Indicate where provided: section/figure legend**	**N/A**
If the study protocol has been pre-registered, provide DOI. For clinical trials, provide the trial registration number OR cite DOI.	N/A	

**Laboratory protocol**	**Indicate where provided: section/figure legend**	**N/A**
Provide DOI OR other citation details if detailed step-by-step protocols are available.	Materials and Methods provides gastruloid culture, immunostaining, mounting/clearing and two-photon imaging. Analysis workflows are documented in the Tapenade repository (README + notebooks).	

**Experimental study design (statistics details) *<br>For in vivo studies: State whether and how the following have been done**	**Indicate where provided: section/figure legend. If it could have been done, but was not, write "not done"**	**N/A**
Sample size determination	No a priori power analysis. Sample sizes (number of gastruloids/images/nuclei) are reported in the Results and/or figure legends; comparisons are primarily descriptive/validation-based.	
Randomisation	not done (not applicable / no group allocation)	

Blinding	not done (not applicable / no group allocation)	
Inclusion/exclusion criteria	not done (not applicable / no group allocation)	

**Sample definition and in-laboratory replication**	**Indicate where provided: section/figure legend**	**N/A**
State number of times the experiment was replicated in the laboratory.	Biological replicates correspond to independent gastruloids; N is indicated in figures/legends for each analysis.	

Define whether data describe technical or biological replicates. Biological replicates are independent gastruloids; nuclei/images are subsamples and may be treated as technical units depending on the analysis.

Ethics:

**Ethics**	**Indicate where provided: section/submission form**	**N/A**
Studies involving human participants: State details of authority granting ethics approval (IRB or equivalent committee(s), provide reference number for approval.	N/A.	N/A
Studies involving experimental animals: State details of authority granting ethics approval (IRB or equivalent committee(s), provide reference number for approval.	N/A (no in vivo vertebrate animal experiments; established mouse ESC lines used).	N/A
Studies involving specimen and field samples: State if relevant permits obtained, provide details of authority approving study; if none were required, explain why.	N/A.	N/A

**Dual Use Research of Concern (DURC)**	**Indicate where provided: section/submission form**	**N/A**
If study is subject to dual use research of concern regulations, state the authority granting approval and reference number for the regulatory approval.	N/A.	N/A

Analysis:

Attrition	Indicate where provided: section/figure legend	N/A
Describe whether exclusion criteria were pre-established. Report if sample or data points were omitted from analysis. If yes, report if this was due to attrition or intentional exclusion and provide justification.	No samples/data points were excluded; all analyses shown report N (gastruloids/images/nuclei) in figure legends.	

Statistics	Indicate where provided: section/figure legend	N/A
Describe statistical tests used and justify choice of tests.	Methods and figure legends report descriptive statistics and validation metrics (e.g., precision/recall/F1/IoU for segmentation; means with SD shading where shown). If any hypothesis tests are used in specific analyses, specify test names and assumptions in Methods/legends.	

Data availability	Indicate where provided: section/submission form	N/A
For newly created and reused datasets, the manuscript includes a data availability statement that provides details for access (or notes restrictions on access).	Data availability statement: datasets used to train nuclei/mitotic-event models and the trained weights are available here https://zenodo.org/records/14748083	
When newly created datasets are publicly available, provide accession number in repository OR DOI and licensing details where available.	https://zenodo.org/records/14748083 Creative Commons Attribution 4.0 International	
If reused data is publicly available provide accession number in repository OR DOI, OR URL, OR citation.		N/A

Code availability	Indicate where provided: section/figure legend	N/A
For any computer code/software/mathematical algorithms essential for replicating the main findings of the study, whether newly generated or re-used, the manuscript includes a data availability statement that provides details for access or notes restrictions.	Code availability statement: Tapenade is available at https://github.com/GuignardLab/tapenade (MIT license). Related napari plugins: https://github.com/GuignardLab/napari-manual-registration; https://github.com/GuignardLab/napari-tapenade-processing; https://github.com/GuignardLab/napari-spatial-correlation-plotter.	
Where newly generated code is publicly available, provide accession number in repository, OR DOI OR URL and licensing details where available. State any restrictions on code availability or accessibility.	https://github.com/GuignardLab/tapenade (MIT license).	
If reused code is publicly available provide accession number in repository OR DOI OR URL, OR citation.	Reused/open-source software includes napari and StarDist3D (and other standard open-source image-analysis dependencies).	

Reporting:

The MDAR framework recommends adoption of discipline-specific guidelines, established and endorsed through community initiatives.

Adherence to community standards	Indicate where provided: section/figure legend	N/A
State if relevant guidelines (e.g., ICMJE, MIBBI, ARRIVE, STRANGE) have been followed, and whether a checklist (e.g., CONSORT, PRISMA, ARRIVE) is provided with the manuscript.	No additional discipline-specific reporting guideline (e.g., CONSORT/PRISMA/ARRIVE) is applicable to this methods/analysis study. MDAR checklist completed; key resources and data/code availability statements provided.	
